# f-treeGC: a questionnaire-based family tree-creation software for genetic counseling and genome cohort studies

**DOI:** 10.1186/s12881-017-0433-4

**Published:** 2017-07-14

**Authors:** Tomoharu Tokutomi, Akimune Fukushima, Kayono Yamamoto, Yasushi Bansho, Tsuyoshi Hachiya, Atsushi Shimizu

**Affiliations:** 1 Department of Clinical Genetics, School of Medicine, Iwate Medical University, Morioka, Iwate 020-8505 Japan; 2 Division of Innovation and Education, Iwate Tohoku Medical Megabank Organization, Iwate Medical University, Shiwa, Iwate 028-3694 Japan; 3 Holonic Systems Ltd., Shiwa, Iwate 028-3441 Japan; 4 Division of Biomedical Information Analysis, Iwate Tohoku Medical Megabank Organization, Iwate Medical University, Shiwa, Iwate 028-3694 Japan

**Keywords:** Pedigree, Family tree, Software, Questionnaire, Family health history, Interview sheet, Genetic counseling, Genome cohort studies, Primary care, Biobank

## Abstract

**Background:**

The Tohoku Medical Megabank project aims to create a next-generation personalized healthcare system by conducting large-scale genome-cohort studies involving three generations of local residents in the areas affected by the Great East Japan Earthquake. We collected medical and genomic information for developing a biobank to be used for this healthcare system. We designed a questionnaire-based pedigree-creation software program named “f-treeGC,” which enables even less experienced medical practitioners to accurately and rapidly collect family health history and create pedigree charts.

**Results:**

f-treeGC may be run on Adobe AIR. Pedigree charts are created in the following manner: 1) At system startup, the client is prompted to provide required information on the presence or absence of children; f-treeGC is capable of creating a pedigree up to three generations. 2) An interviewer fills out a multiple-choice questionnaire on genealogical information. 3) The information requested includes name, age, gender, general status, infertility status, pregnancy status, fetal status, and physical features or health conditions of individuals over three generations. In addition, information regarding the client and the proband, and birth order information, including multiple gestation, custody, multiple individuals, donor or surrogate, adoption, and consanguinity may be included. 4) f-treeGC shows only marriages between first cousins via the overlay function. 5) f-treeGC automatically creates a pedigree chart, and the chart-creation process is visible for inspection on the screen in real time. 6) The genealogical data may be saved as a file in the original format. The created/modified date and time may be changed as required, and the file may be password-protected and/or saved in read-only format. To enable sorting or searching from the database, the file name automatically contains the terms typed into the entry fields, including physical features or health conditions, by default. 7) Alternatively, family histories are collected using a completed foldable interview paper sheet named “f-sheet”, which is identical to the questionnaire in f-treeGC.

**Conclusions:**

We developed a questionnaire-based family tree-creation software, named f-treeGC, which is fully compliant with international recommendations for standardized human pedigree nomenclature. The present software simplifies the process of collecting family histories and pedigrees, and has a variety of uses, from genome cohort studies or primary care to genetic counseling.

**Electronic supplementary material:**

The online version of this article (doi:10.1186/s12881-017-0433-4) contains supplementary material, which is available to authorized users.

## Background

Genealogical information is critical for accurate genetic diagnosis in clinical genetics. In 1995, the National Society of Genetic Counselors [1] introduced a standardized description method for presenting a family tree in genetic counseling, which was revised in 2008 to the current version [2]. Typically, pedigree charts are manually created via face-to-face personal interviews held on an individual basis. However, pedigree chart creation requires graphical skills and specialized knowledge of clinical genetics, and is thus a time- and labor-intensive process. A large amount of genetic data has been collected from numerous large-scale studies conducted in recent years, such as genome-cohort studies. The availability of accurate genealogical information is essential for performing comprehensive analysis of polymorphisms and associated diseases for use in genetic counseling, research, and diagnosis. Given the large amount of genetic information required and collected, it is currently not possible to create pedigree charts using the traditional method through interviews at a single recruiting event by an expert.

The Tohoku Medical Megabank (TMM) project [3] aims to restore community medical services that were negatively affected by the Great East Japan Earthquake, and to create a next-generation personalized healthcare system by conducting large-scale genome-cohort studies involving three generations of local residents in the disaster-stricken areas [4]. Specifically, we collected medical and genomic information, including family health history, for developing a biobank to be used in the planned healthcare system.

In this project, we designed a questionnaire-based pedigree-drawing software program named “f-treeGC”, which enables even less-experienced medical practitioners to accurately and rapidly collect genealogical information and create pedigree charts, in full compliance with international standards [2].

## Implementation

### Programming

f-treeGC is written in ActionScript 3.0, and may be run on Adobe AIR, which is a cross-platform runtime system.

### Operating environment

f-treeGC is supported by both Windows (Windows 7, 8, and 10) and Macintosh (operating system (OS) X). Adobe AIR Runtime [5] must be installed before installation and use of f-treeGC, and Adobe Reader DC [6] is required for the printing function. Via these programs, the f-treeGC air file may be opened to install the software. The f-treeGC software program is available for use, at no monetary cost, at the Iwate Medical University Hospital website (http://www.iwate-med.ac.jp/hospital/clinics/medical/m26/).

## Results

The method for the creation of pedigree charts is described in the following sections.

### Confirmation of whether or not the client has a child

**Fig. 1:**
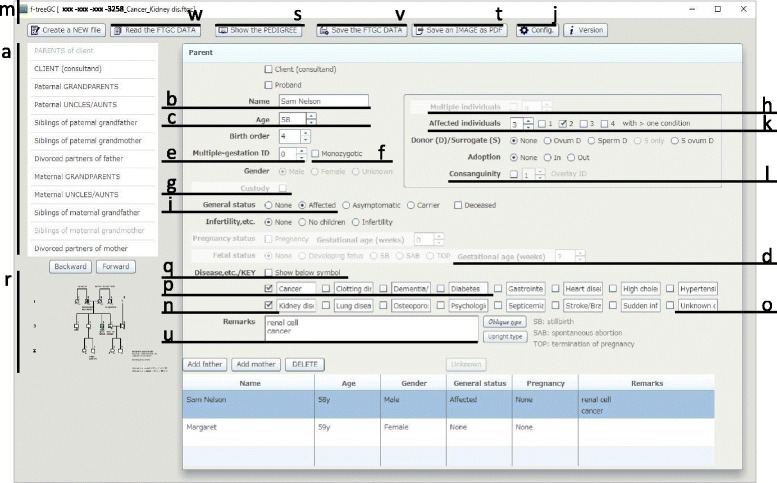
Information entry screen of f-treeGC; information for three generations is required to create the pedigree (*a*). The genealogical information requested includes name (*b*, *u*), age (*c*, *d*), gender, general status [affected (*i*, *j*, *k*), asymptomatic/presymptomatic carrier, carrier, or deceased], infertility status, pregnancy status, fetal status, and disease status (*m*, *n*, *o*, *p*, *q*, *t*) of individuals belonging to three generations. In addition, client, proband and birth order information, including multiple gestation (*e*, *f*), custody (*g*), multiple individuals (*h*), donor or surrogate, adoption, and consanguinity (*l*) information may be inserted. The chart-creation process is visible for inspection on the lower left side of the f-treeGC screen in real time (*r*). A multiple-choice questionnaire for genetic information is filled out by an interviewer or the client; then, f-treeGC automatically creates a pedigree chart (*r*, *s*, *t*, *v*, *w*)

**Fig. 2:**
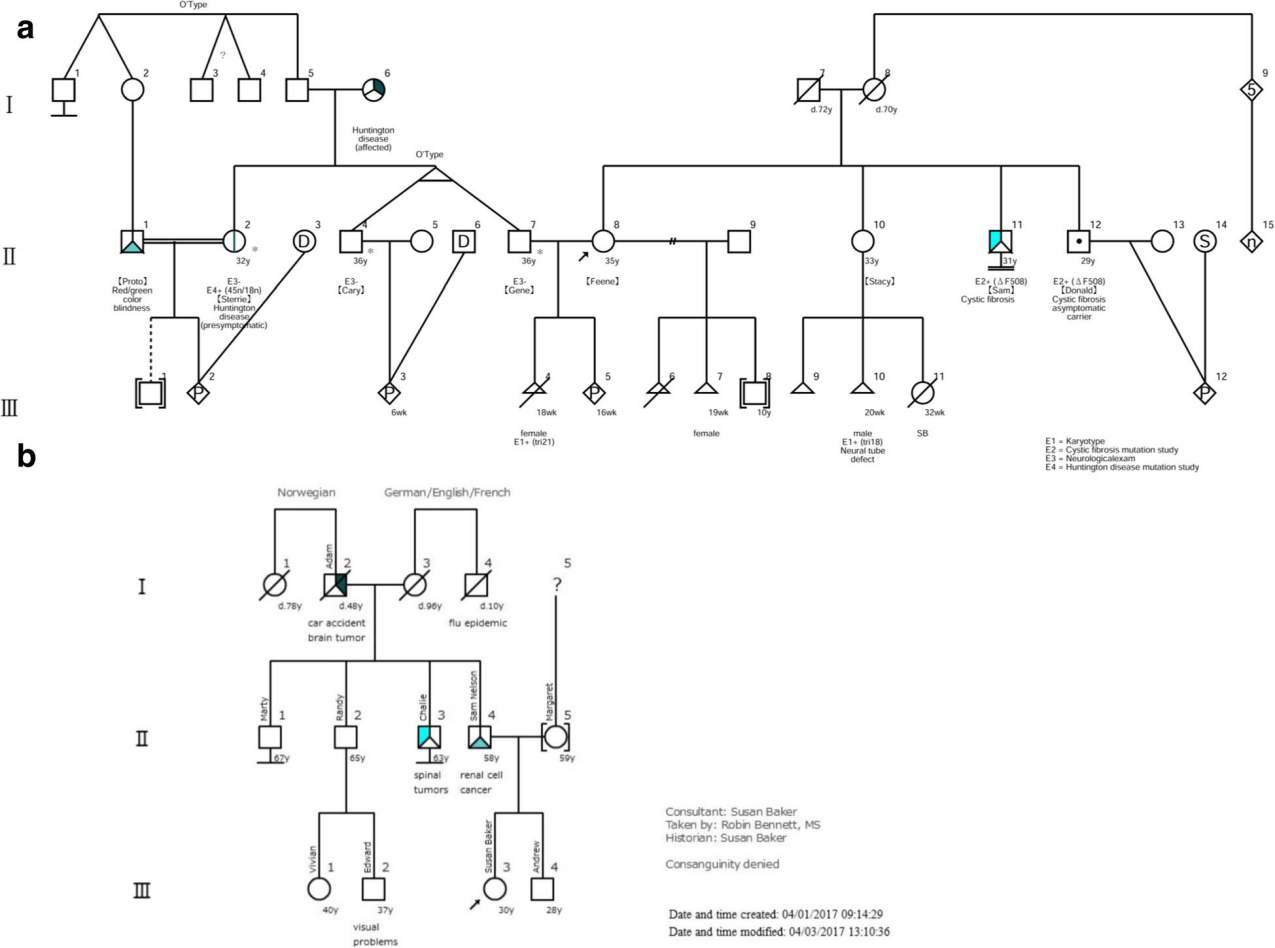
Fictitious pedigrees created by f-treeGC. **a** Fictitious ultimate pedigree, **b** A hypothetical pedigree representative of a family with von Hippel-Lindau syndrome shown in Fig. 1, partially modified from Bennett et al. [1, 7]. The clients are included in the 2nd generation (**a**) or in the 3rd generation (**b**) upon confirmation at system startup

At system startup, the client is prompted to provide required information on the presence or absence of children (Additional file 1a). f-treeGC is capable of creating a pedigree that includes three generations (Fig. 1a). Couples with offspring are included in the 2nd generation of the family tree (Fig. 2a, Additional file 2), whereas clients with no children are included in the 3rd generation (Figs. 1a and 2b, Additional file 3). A representative correspondence table is shown in Table 1.

**Table 1:** Correspondence table of f-treeGC

Client has a child	Client has NO children
Client (consultand) couple	Parents of client
Children of client couple	Client (consultand)
Husband’s (his) parents	Paternal grandparents
His siblings	Paternal uncles/aunts
His paternal uncles/aunts	Siblings of paternal grandfather
His maternal uncles/aunts	Siblings of paternal grandmother
His divorced partners	Divorced partners of father
Wife’s (her) parents	Maternal grandparents
Her siblings	Maternal uncles/aunts
Her paternal uncles/aunts	Siblings of maternal grandfather
Her maternal uncles/aunts	Siblings of maternal grandmother
Her divorced partners	Divorced partners of mother

### Multiple-choice questionnaire

The genealogical information requested includes name, age, gender, general status (affected, asymptomatic/presymptomatic carrier, carrier, or deceased), infertility status, pregnancy status, fetal status, and health status (occurrence of any diseases) of individuals in the three generations. In addition, information regarding client and proband, as well as birth order information such as multiple gestation, custody, multiple individuals, donor or surrogate, adoption, and consanguinity may be included (Fig. 1).

By default, the “Name” field refers to the type of relationship such as father or mother. The user should delete the relationship name before inputting the relevant name (Fig. 1b). In the “Age” box (Fig. 1c) and the “Gestational age (weeks)” box (Fig. 1d), the user may select “In blank”, “? (unknown)”, or the relevant number. Inputting the same number of individuals in the “Multiple-gestation ID” (Fig. 1e) indicates multiple gestation or pregnancy with multiple fetuses. The “Monozygotic” box (Fig. 1f) is for identical twins (pregnancy). The “Custody” box (Fig. 1g) is for the position of a break in a relationship line between divorced partners, and indicates the parent(s) with primary responsibility for the children following divorce. For multiple individuals, users may select “n (unknown)” or the relevant number(s) after checking the “Multiple individuals” box (Fig. 1h). The “Affected” button of general status (Fig. 1i) is for affected individuals, and users may set a key color for the affected status in the configuration (Fig. 1j). For affected individuals with two or more conditions, the user may check for the conditions (Fig. 1k). Considering the diversity in color perception and to enable distinction in subsequent black-and-white photocopies, f-treeGC shows multiple conditions using a color of a similar shade (Additional file 1b).

### A printed paper version of the questionnaire

**Fig. 3:**
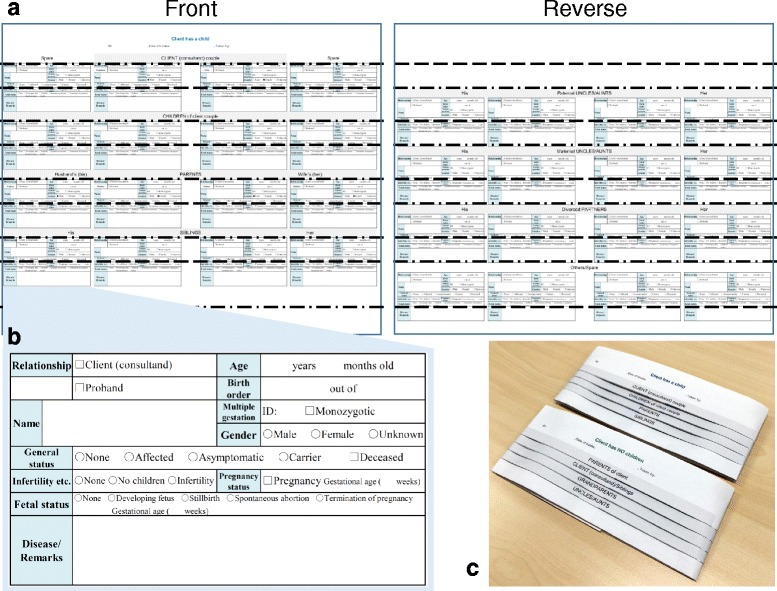
Foldable interview sheet named “f-sheet”. **a** Large sheet, **b** gathered interview sheets for each individual, and **c** accordion-folded sheet. In diagram (**a**), the dashed-dotted lines, “-・-・-・-・-”, and broken lines, “- - - - -”, show the mountain fold and the valley fold, respectively

Alternatively, family health histories may be collected using the foldable interview sheet named “f-sheet” (Fig. 3, Additional file 4), which is a printed paper version of the questionnaire in f-treeGC. The f-sheet provides an overview of genetic relationships between families according to the manner in which the sheet is folded or developed. For example, the vertical line of the folded f-sheet corresponds to the left panel of the f-treeGC (Fig. 1a). Filling out a multiple-choice questionnaire on genetic information is easier for a medical practitioner with poor digital literacy. A skilled data entry clerk may subsequently input family health histories into f-treeGC from the f-sheet as a bundle.

### Overlay function for consanguinity

**Fig. 4:**
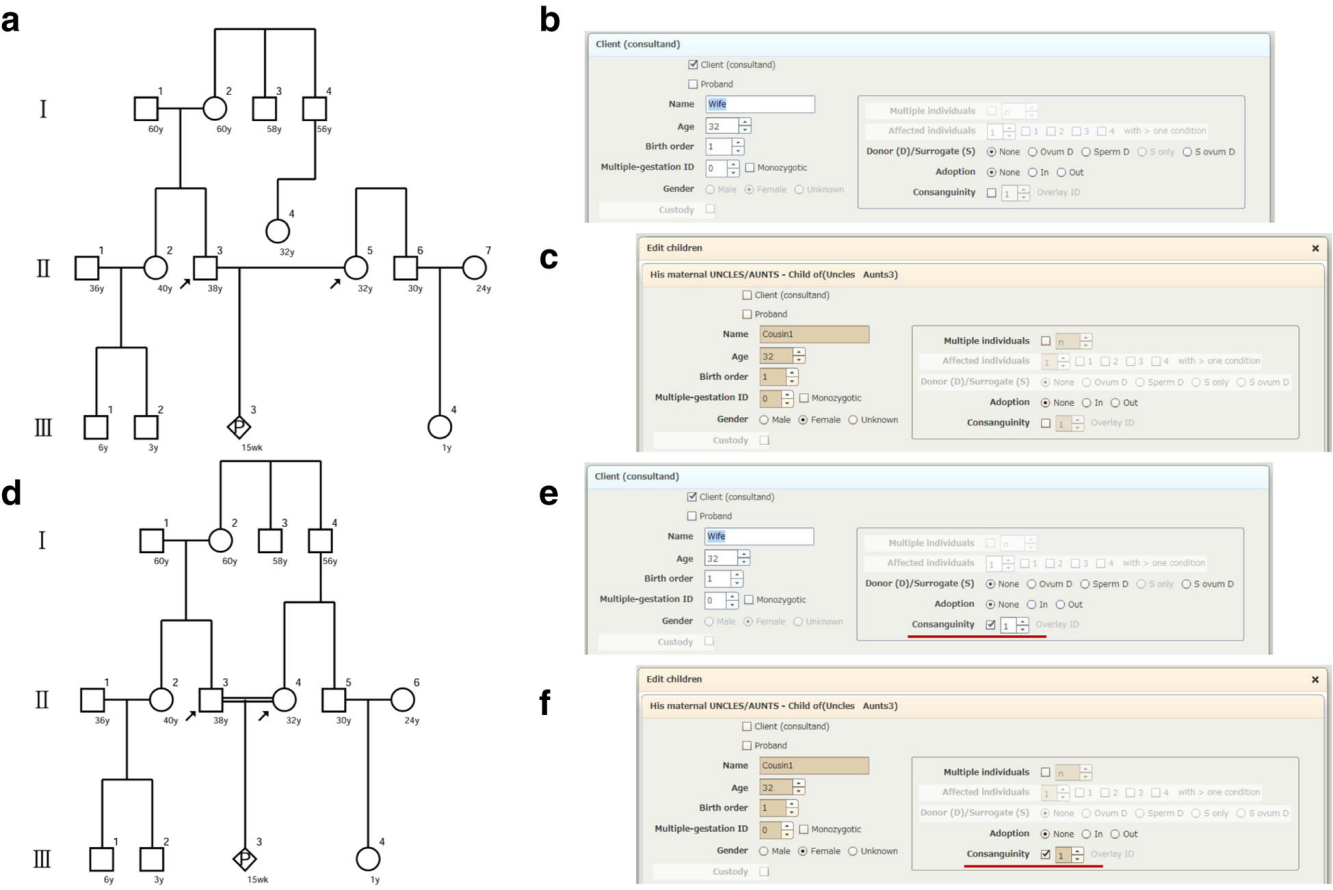
Overlay function of f-treeGC for consanguinity; a pedigree before (**a**) and after (**d**) overlay; the interview sheets of the wife (**b**, **e**) and a first cousin (**c**, **f**)

With respect to consanguinity, f-treeGC shows only marriages between first cousins using the overlay function. The “Consanguinity” box should be checked and the same “Overlay ID” should be entered for this function to be effective (Figs. 1l and 4, Additional file 5).

### Entry fields and keys for physical features or diseases/conditions for genome cohort studies

By default, the file name automatically contains the first ten letters (Fig. 1m) that are typed into the sixteen entry fields of the “Disease, etc./KEY,” such as disease name (Fig. 1n), and for which the box is checked (Fig. 1o), to enable sorting or searching from the database. For example, the preliminary physical features entered are hair, ears, eyes, nose, philtrum, oral region, neck, hands/feet, chest, skin, abdominal, genitalia, and skeletal [7]. Diseases or conditions included in the My Family Health Portrait tool [8] include cancer, clotting disorder, dementia/Alzheimer’s disease, diabetes, gastrointestinal disorder, heart disease, high cholesterol, hypertension, kidney disease, lung disease, osteoporosis, psychological disorder, septicemia, stroke/brain attack, sudden infant death syndrome, and unknown disease (Fig. 1o). The first four out of sixteen entry fields may also be used as keys related to the multiple conditions of an affected individual (Fig. 1k, p). The “Show below symbol” box is for showing the entry below each symbol, only if the box on the left of the entry field is checked (Fig. 1q).

### Pedigree chart creation

f-treeGC automatically creates a pedigree chart; the chart-creation process is visible for inspection on the screen in real time (Fig. 1r). The “Show the PEDIGREE” button (Fig. 1s) at the top of the screen displays a larger chart (Fig. 2b). The user may additionally edit a pedigree chart to visualize the larger chart with multiple displays.

A context menu appears when the family tree is right-clicked. The user may select “Add comment,” right-click on the comment box, and then type the appropriate text. Holding down the left mouse button allows the comment in the family tree to be moved and placed accordingly. The user may delete the comment, if required, by selecting “Delete comment”. By default, a pedigree chart does not include the name input (Fig. 1b, t) for privacy. The user may see names by selecting “Show names” in the context menu. To print or save an image of the pedigree, including names or other health conditions, the user may capture a screen shot (Fig. 2b, Additional file 3), or input the names manually in the “Remarks” box (Figs. 1u and 2a, Additional file 2).

### Saving and reading the data

The genealogical information data may be saved as the original format file (FTGC file) by clicking the “Save the FTGC DATA” button (Fig. 1v), and shared between computers that have f-treeGC installed. The created/modified date and time may be changed as necessary before saving the data (Additional file 1c). In addition, the image of the pedigree chart may be saved in PDF format by clicking the “Save an IMAGE as PDF” button (Figs. 1t and 2a). The file may be password-protected and/or saved in a read-only format (Additional file 1c). A saved file may be read by clicking the “Read the FTGC DATA” button (Fig. 1w) at the top of the screen and selecting the file.

### Comparison of the pedigree symbols used

A comparison of the pedigree symbols used in f-treeGC with those of several existing tools [8–11] is shown in Table 2. f-treeGC complies fully with the international recommendations of standardized human pedigree nomenclature [2], including common pedigree symbols, pedigree lines, assisted reproductive technology symbols, and pedigree symbols of genetic evaluation/testing information (Additional file 6).

**Table 2:** Comparison of the pedigree symbols used

Pedigree software packages	f-treeGC	PedigreeXP	Progeny Free Online Pedigree Tool	Genial Pedigree Draw	My Family Health Portrait
Version	1.01	2.1.0.169	Not described	2.0	3.4
Standard pedigree symbols^a^					
Name of proband/consultand	Yes	Yes	Yes	Yes	No
Family names	Yes	Yes	Yes	Yes	Yes
Historian	Yes^b^	No	Yes^b^	No	No
Date of intake/update	Yes	No	No	No	Yes
Reason for taking pedigree	Yes^b^	No	Yes^b^	No	No
Ancestry of both sides of the family	Yes	Yes	Yes	Yes	No
Age	Yes	Yes	Yes	Yes	No
Generation numbered with Roman numerals I, II, III, etc.	Yes	Yes	Yes^b^	Yes^b^	No
Individual numbered 1, 2, 3, etc.	Yes	Yes	Yes^b^	Yes^b^	No
Individual	Yes	Yes	Yes	Yes	Yes
Affected individual	Yes	Yes	Yes^d^	Yes	Yes
Affected individual (> one condition)	Yes^c^	Yes^c,d^	Yes	Yes	No
Multiple individuals, number known	Yes	Yes	Yes	Yes	No
Multiple individuals, number unknown	Yes	Yes	Yes	Yes	No
Deceased individual	Yes	Yes	Yes	Yes	Yes
Consultand	Yes	No	No	Yes	No
Proband	Yes	Yes^d^	Yes^d^	Yes	No
Stillbirth (SB)	Yes^b^	No	No	No	No
Pregnancy (P)	Yes	Yes	Yes	Yes	No
Spontaneous abortion (SAB)	Yes	Yes	Yes^d^	Yes	No
Affected SAB	Yes	Yes	Yes^d^	No	No
Termination of pregnancy (TOP)	Yes	Yes	No	Yes	No
Affected TOP	Yes	Yes	No	No	No
Ectopic pregnancy	Yes^b^	Yes^b^	No	Yes	No
Relationship line	Yes	Yes	Yes	Yes	Yes
A break in relationship line	Yes	Yes	Yes	Yes	No
Divorced partner	Yes^e^	Yes	Yes	Yes	No
Consanguinity	Yes	Yes	Yes	Yes	No
Multiple gestation, monozygotic	Yes	Yes	Yes	Yes	No
Multiple gestation, dizygotic	Yes	Yes	Yes	Yes	No
Multiple gestation, unknown	Yes^b^	No	Yes^b^	No	No
Multiple gestation, trizygotic	Yes	Yes	Yes	Yes	No
Family history not available/known for individual	Yes	No	No	No	No
No children by choice or reason unknown	Yes	No	Yes	Yes^d^	No
Infertility	Yes	No	Yes	Yes^d^	No
Adoption	Yes	Yes	Yes	Yes	Yes^d^
Ovum or sperm donor	Yes	No	No	Yes	No
Surrogate (gestational carrier)	Yes	No	No	Yes	No
Evaluation	Yes^b^	Yes^b^	Yes	Yes^b^	No
Carrier	Yes	Yes^d^	Yes^f^	Yes	No
Asymptomatic/presymptomatic carrier	Yes	No	No	Yes	No
Uninformative study	Yes^b^	Yes^b^	Yes^b^	Yes^b^	No
Affected individual with positive evaluation	Yes^b^	Yes^b^	Yes	Yes^b^	No

^a^Bennett et al., 2008 [2]

^b^By using “Remarks”, “Comment”, “Notes”, or “Annotated Text”

^c^Up to four conditions

^d^Different symbols used from the recommended common pedigree symbols

^e^Limited to three individuals (consultand couple with children, or parents of consultand without children)

^f^By using “Apply symbols”

### System features and functions of f-treeGC as a family history collection tool

A well-designed online family history tool from the US Surgeon General, called My Family Health Portrait, is used for the collection and storage of family history data [11, 12]. In addition, the MeTree software program developed by the Duke Center for Applied Genomics and Precision Medicine enables collection of family health history and provides clinical decision-making support for more than 30 conditions such as cancer, cardiovascular diseases, liver, and diabetes [12, 13]. The Global Alliance for Genomics and Health (GA4GH) provides the GA4GH family history collection and clinical decision support tool inventory, and is open for submission. The information collected by f-treeGC is shown in Table 3 and in Additional file 7 derived from the submission form of the GA4GH family history tools catalog [12].

**Table 3:** f-treeGC information

Provenance	Academically developed
Tool URL	http://www.iwate-med.ac.jp/hospital/clinics/medical/m26/
Target clinical population	Primary care and specialty
Family history information - source	Multiple sources
Family history information - data format	Both structured and free text
Family history information - analysis	Manual
Result recipients	Clinician
Data storage	Store in the original format file
Discrete data integration readiness	Application programing interfaces (API)
Consent documentation	Implied consent only
System features - platforms	Windows and Macintosh
	Built in Adobe Air
System features - architecture	Stand-alone
System features - security	Password security protection
	Read-only mode
Standard pedigree symbols	Fully compliant
Other functions	Custody
	Pregnancy with twins or multiples

### Verification of the software and interview sheet

To verify that f-treeGC enables users without specialized knowledge of clinical genetics and graphical skills to easily create medical pedigrees, we provided nine subjects (six nurses and three clerks) with two scenarios (D, Duchenne muscular dystrophy; P, phenylketonuria) of fictitious family histories (Additional file 8), and compared the pedigrees obtained by f-treeGC (Additional file 9) with those derived manually. The pedigrees were scored according to a system of allocation points (Additional file 8) based on the international standard [2] to examine the performance and usability of f-treeGC. The creation time was indefinite, and we divided the trees into two groups with different orders of scenarios applied. We used Windows 7 as the OS for this test.

To verify that f-sheet improves the user experience for data input to f-treeGC, we provided 47 high school students from one high school (males, *N* = 28; females, *N* = 19; age range, 15–16 years; grade, the first year) with the software and data for two scenarios. Students were randomly assigned to two groups: students of one group created pedigrees for both scenarios (Additional file 8) using f-treeGC without f-sheet (group TT; males, *N* = 17; females, *N* = 6). Students of the other group first created pedigrees for scenario D using f-treeGC without f-sheet, and then created pedigrees for scenario P using f-treeGC with a completed f-sheet (group TS; males, *N* = 11; females, *N* = 13). The pedigrees with or without f-sheet were scored using our points allocation system (Additional file 8) based on the international standard [2]; then, the scores were compared to examine the efficacy and usability of f-sheet. The creation time was indefinite. The OS used for this purpose was Windows 7.

The Wilcoxon signed-rank test and Mann-Whitney U-test were performed for statistical analyses, using Statcel4 software (OMS Ltd. Publishing, Saitama, Japan). Significance was set at *p* < 0.05.

The family trees obtained using f-treeGC had higher scores than those that were manually created (*p* < 0.001) (Table 4). Moreover, the input time and family tree scores of trees created using f-treeGC were not affected by the difference in scenario content, order of application, or the qualifications of each user (Table 4). Furthermore, the scores of the family trees created using f-treeGC with a completed f-sheet were higher than those created using f-treeGC without f-sheet (*p* < 0.01) (Table 5).

**Table 4:** Comparison of pedigree scores and creation time for each factor

Factors	Median (Range)				*P* value		*N*	Statistics
Method (manual method vs. f-treeGC software)	Manual		f-treeGC					Wilcoxon signed-rank sum test
Score (%) of both scenarios	59.5	(23.7)	86.4	(25.8)	0.00019	***	18	
Score (%) of scenario D	58.8	(14.7)	86.8	(16.2)	0.00769	**	9	
Score (%) of scenario P	60.2	(23.7)	86.0	(25.8)	0.00742	**	9	
Order of scenarios (D to P vs. P to D)	D to P		P to D					Mann-Whitney U-test
Score (%) of scenario D by the manual method	55.9	(11.8)	61.0	(14.7)	0.32312		5 × 4	
Time (sec/individuals) of scenario D by the manual method	42.5	(18.8)	25.9	(12.5)	0.01390	*	5 × 4	
Score (%) of scenario P by the manual method	60.2	(11.8)	57.5	(23.7)	1.00000		5 × 4	
Time (sec/individuals) of scenario P by the manual method	26.7	(16.3)	28.8	(33.3)	0.32516		5 × 4	
Score (%) of scenario D by f-treeGC	83.8	(16.2)	87.5	(13.2)	0.61980		5 × 4	
Time (sec/individuals) of scenario D by f-treeGC	65.0	(49.4)	61.9	(23.4)	0.46243		5 × 4	
Score (%) of scenario P by f-treeGC	86.0	(25.8)	87.6	(8.5)	1.00000		5 × 4	
Time (sec/individuals) of scenario P by f-treeGC	46.7	(25.4)	51.5	(44.3)	0.32719		5 × 4	
Order of scenarios (1st vs. 2nd)	1st		2nd					Wilcoxon signed-rank sum test
Score (%) by the manual method	55.9	(23.7)	60.3	(14.7)	0.44127		9	
Time (sec/individuals) by the manual method	40.0	(37.5)	26.7	(19.2)	0.01086	*	9	
Score (%) by f-treeGC	83.9	(18.9)	86.8	(25.8)	0.67840		9	
Time (sec/individuals) by f-treeGC	59.2	(65.6)	50.4	(38.5)	0.13864		9	
Qualification (nurse vs. clerk)	Nurse		Clerk					Mann-Whitney U-test
Score (%) by the manual method	61.0	(23.7)	54.1	(17.2)	0.13339		12 × 6	
Time (sec/individuals) by the manual method	30.8	(40.8)	26.3	(45.4)	0.67310		12 × 6	
Score (%) by f-treeGC	86.4	(17.2)	86.8	(25.8)	0.85103		12 × 6	
Time (sec/individuals) by f-treeGC	57.1	(53.1)	39.4	(47.9)	0.18955		12 × 6	

*p < 0.05

**p < 0.01

***p < 0.001

**Table 5:** Comparison of pedigree scores using f-treeGC with or without f-sheet

Factors	Median (Range)				*P* value		*N*	Statistics
Group (TT vs. TS)	TT		TS					Mann-Whitney U-test
Score (%) of scenario D	89.7	(10.3)	87.5	(26.5)	0.0196	*	23 × 24	
Score (%) of scenario P	88.8	(24.5)	92.3	(17.3)	0.0109	*	23 × 24	
Scenario (D vs. P)	D		P					Wilcoxon signed-rank sum test
Score (%) of group TT	89.7	(10.3)	88.8	(24.5)	0.6922		23	
Score (%) of group TS	87.5	(26.5)	92.3	(17.3)	0.0018	**	24	

*p < 0.05

**p < 0.01

## Discussion

In the present study, we report the development of f-treeGC, a free stand-alone application built as a cross-platform runtime system. f-treeGC is capable of automatically creating a medical family tree compliant with international standards [2] by filling out available family tree information on a medical interview sheet (Fig. 1). Family histories are entered as both structured data and free text by the clinician or data entry clerk, and collected from patients or through f-sheet, which is a printed paper version of the questionnaire in f-treeGC (Fig. 3, Additional file 4). The family history data are stored in a computer in the original format file. f-treeGC may be used for collecting family health histories and creating pedigrees for individuals participating in situations such as primary care, genetic counseling, or genome cohort studies. The targeted clinical populations are recipients of primary and specialty health care facilities.

f-treeGC simplifies the process of creating pedigrees by confirming whether the client has offspring at system startup (Table 1, Additional file 1a) and by using the overlay function for confirming consanguinity (Fig. 4). Here, we show that f-treeGC, which is fully compliant with international recommendations for standardized human pedigree nomenclature (Table 2), is highly useful for creating pedigree charts for applications in genetic counseling.

However, the present study is not without limitations. As f-treeGC is only capable of creating a pedigree up to three generations, this software is currently unsuitable for creating large pedigrees. There are no auxiliary input functions for medical terms, pedigree-overlay function, nor a calculator for determining disease risk. f-treeGC is not adapted for compliance with Health-Level 7 (HL7) standards. Although numerous health and medical conditions exist [13], f-treeGC is limited to only sixteen medical conditions per person.

Low quality of family history data collected presents a challenge in pedigree analysis [14]. Before collecting family health histories, users should guide patients regarding what to inquire of relatives, as the amount and accuracy of the family history is limited. The Iwate Tohoku Medical Megabank Organization conducts genetics workshops mentored by medical geneticists or genetic counselors to highlight the importance of family health history before recruiting participants for cohort studies of the TMM project.

Family history, the ultimate genetic tool [15], is the most cost-effective and well known “genetic test” in clinical practice today [16]. However, recording family trees according to standard recommendations generally requires knowledge of graphical interfaces and clinical genetics [7]. In 2016, the National Human Genome Research Institute (NHGRI) convened a Family Health History Tool Meeting at the National Institute of Health (NIH) for identifying and sharing successful approaches to using family health history tools, and for identifying unresolved issues and potential solutions that may be addressed by policy, research, and/or collaborative efforts. The removal of barriers to health equality in populations with low levels of literacy, and exploration/expansion additional technological approaches for family health history collection was discussed in this meeting [14].

Six years have passed since the Great East Japan Earthquake and Tsunami. However, health and medical services has not been fully restored to date. The TMM project initiated two prospective cohort studies in the Miyagi and Iwate prefectures, which include the disaster-stricken areas: a population-based adult cohort study, in which 80,000 participants were recruited, and a birth and three-generation cohort study, in which 70,000 participants, included fetuses and their parents, siblings, grandparents, and extended family members, were recruited [4]. Collection of significant numbers of family health histories by conventional pedigree-drawing software programs is challenging in these regions owing to the lack of good internet service and personal computers. We used f-treeGC for genetic counseling at our institution, collecting approximately 100 patient histories and corresponding data, which would have taken a genetic counselor around twenty minutes in a clinical setting. In contrast, the software took about one minute per person to input two clinical scenarios at the verification experiment (Table 4). Since the use of f-treeGC in combination with f-sheet simplifies the process of collection of many numerous health histories and pedigrees (Table 5), its application is not limited to heredity clinics, but also to large-scale genome-cohort studies that handle large amounts of genetic information obtained through interviews at a single recruiting event.

The main advantages of f-treeGC are collection of several family histories for large-scale cohort studies in a short period of time, easing the burden of collection of genealogical information and creation of pedigree charts in remote medical practice by less experienced medical practitioners. In addition, the present tool facilitates online genetic counseling owing to its complete compliance with the international recommendations for standardized human pedigree nomenclature (Table 2).

Public awareness regarding the basic principles of genetics should be considered for the improvement of public health. Familial/pedigree information is valuable for variant filtering in high-throughput sequencing studies [17, 18]. Molecular approaches for the identification of disease-associated genes generally begin with pedigree-based methods, including positional cloning and founder gene approaches, prior to the use of pedigree-independent methods such as candidate gene approaches and genome-wide association studies [19]. With the recent explosion in whole-genome sequencing, linkage analysis has emerged as an important and powerful analytical method for the identification of genes involved in disease etiology, often in conjunction with whole-genome sequencing filtering approaches [20]. From this perspective, f-treeGC is a useful tool, not only for facile and accurate pedigree analysis, but also for conveniently collecting numerous family histories and pedigrees simultaneously. In future, we aim to add a calculator function for determining disease risk, an auxiliary input function for medical terms, a search function for family health conditions from free text, an adaption for HL7, and a pedigree-overlay function to the present f-treeGC software.

## Conclusions

The f-treeGC software enables collection of family health history and automatically creates a medical family tree simply by filling out family tree information on a medical interview sheet, or by inputting the information in the questionnaire directly from the f-sheet.

## Availability and requirements

**Project name:** TMM project

**Project home page:** http://www.amed.go.jp/en/program/list/04/01/042.html

**Operating systems:** Windows and Macintosh

**Programing language:** ActionScript 3.0

**Licence:** f-treeGC is a non-copylefted software, and is copyrighted by the Iwate Medical University and Holonic Systems, Ltd. The source code is not available.

## Additional files

Additional file 1: Dialog boxes of f-treeGC (a) Confirmation of whether or not the client has a child at system startup, (b) Configuration of the color for affected individuals, (c) File attribute setting for changing the created/modified date and time, setting a password, and converting to read-only. (PPTX 141 kb)

Additional file 2: A pedigree file of Fig. 2a; fictitious ultimate pedigree, partially modified from Bennett et al. [1, 7]. (FTGC 20 kb)

Additional file 3: A pedigree file of Fig. 2b; a hypothetical pedigree representative of a family with von Hippel-Lindau syndrome, partially modified from Bennett et al. [1, 7]. (FTGC 9 kb)

Additional file 4: f-sheet; a printed paper version of the questionnaire in f-treeGC. (XLSX 57 kb)

Additional file 5: A pedigree file of Fig. 4d; with respect to consanguinity, f-treeGC shows only marriages between first cousins using the overlay function. (FTGC 8 kb)

Additional file 6: List of symbols used in f-treeGC; f-treeGC fully complies with the international recommendations of standardized human pedigree nomenclature [2]. (PDF 8 kb)

Additional file 7: Current family history collection tools and f-treeGC; partially modified from GA4GH family history collection and clinical decision support tool inventory 6–9-16 v4.1 by Clinical Working Group of the Global Alliance for Genomics and Health. (XLSX 18 kb)

Additional file 8: Two scenarios for the creation of pedigrees; scenario D (Duchenne muscular dystrophy) and scenario P (phenylketonuria). Any resemblance to real persons and pedigrees, living or dead, is purely coincidental. (XLSX 12 kb)

Additional file 9: Model pedigrees of scenarios outlined (a) Scenario D, (b) scenario P. (PPTX 389 kb)

## Abbreviations

GA4GH: Global Alliance for Genomics and Health
TMM project: Tohoku Medical Megabank project
