# Supplementary figures and images for: f-treeGC: a questionnaire-based family tree-creation software for genetic counseling and genome cohort studies
Source: BMC Med Genet. 2017 Jul 14;18:71. doi: 10.1186/s12881-017-0433-4 (PMC5512935; doi:10.1186/s12881-017-0433-4)

## Slide 1
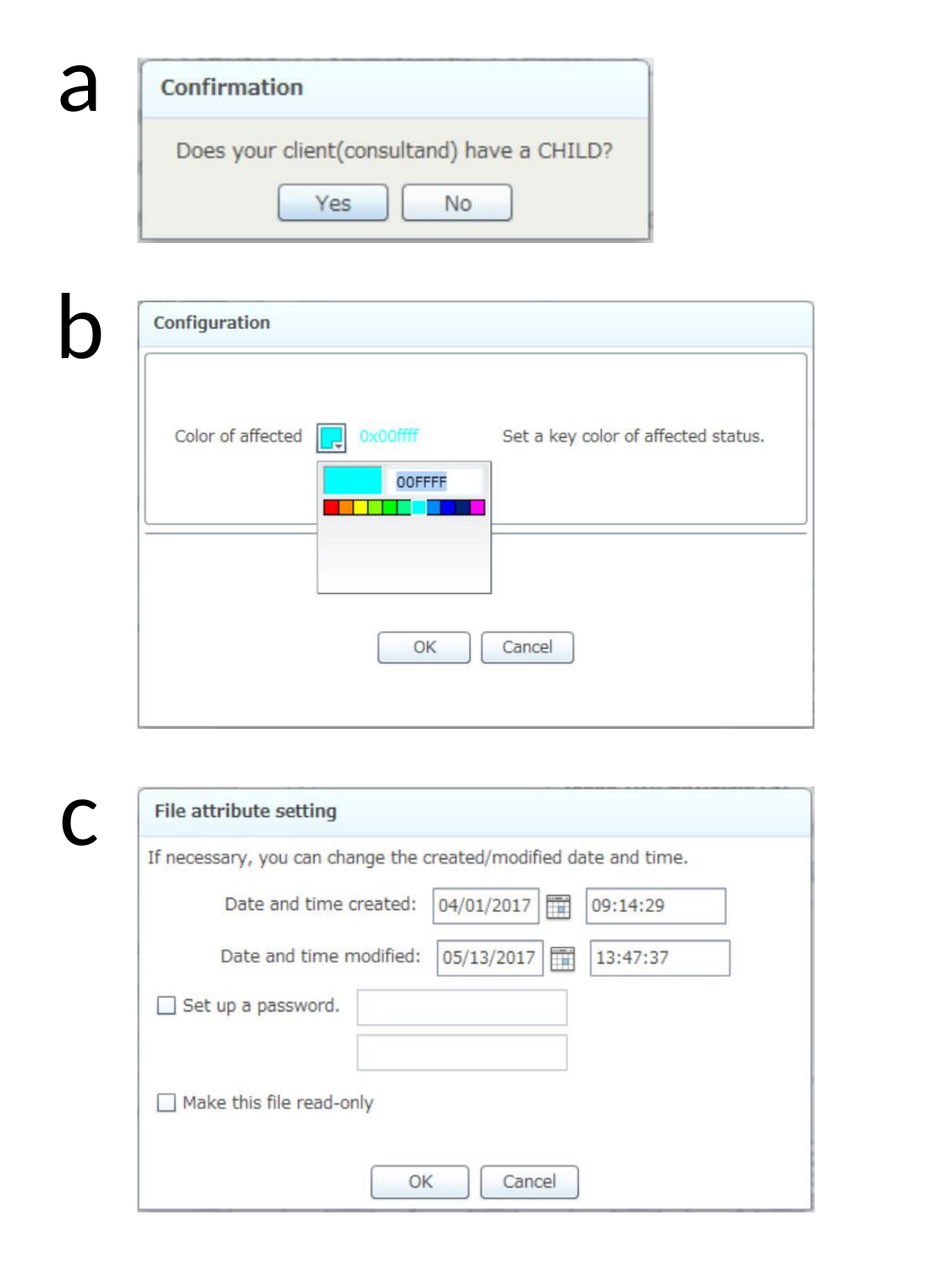

a
b
c

Supplement: Supplementary file 1 — Dialog boxes of f-treeGC (a) Confirmation of whether or not the client has a child at system startup, (b) Configuration of the color for affected individuals, (c) File attribute setting for changing the created/modified date and time, setting a password, and converting to read-only. (PPTX 141 kb) [file 12881_2017_433_MOESM1_ESM.pptx]

## Slide 1
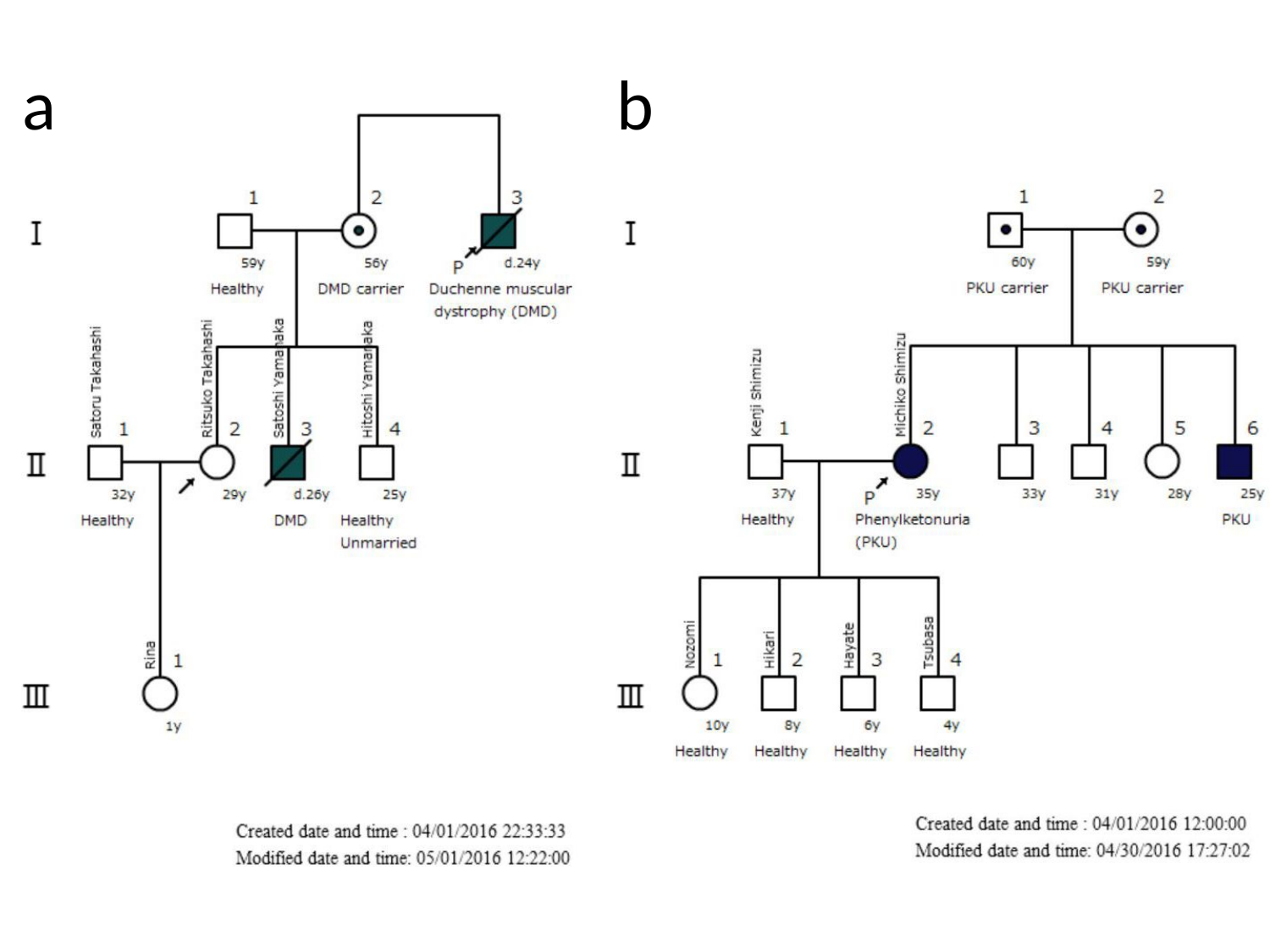

a
b

Supplement: Supplementary file 9 — Model pedigrees of scenarios outlined (a) Scenario D, (b) scenario P. (PPTX 389 kb) [file 12881_2017_433_MOESM9_ESM.pptx]
