# Supplementary material for: f-treeGC: a questionnaire-based family tree-creation software for genetic counseling and genome cohort studies
Source: BMC Med Genet. 2017 Jul 14;18:71. doi: 10.1186/s12881-017-0433-4 (PMC5512935; doi:10.1186/s12881-017-0433-4)
Supplement: Supplementary file 6 — List of symbols used in f-treeGC; f-treeGC fully complies with the international recommendations of standardized human pedigree nomenclature [2]. (PDF 8 kb) [file 12881_2017_433_MOESM6_ESM.pdf]

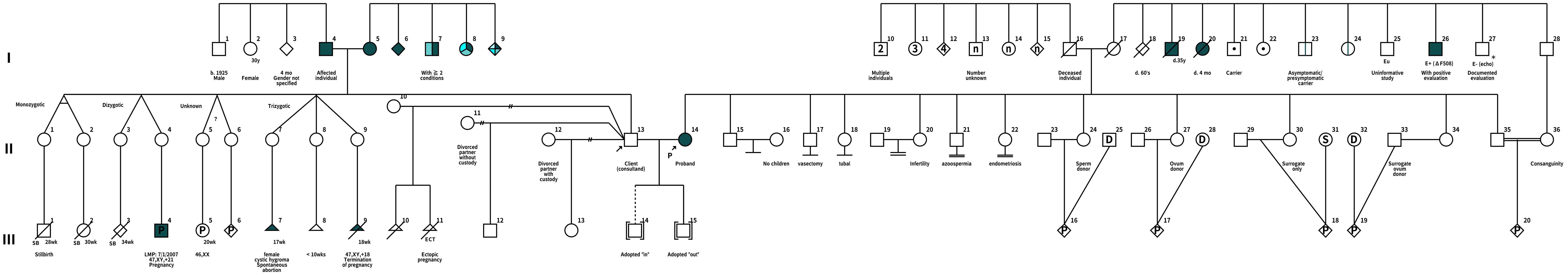

Date and time created: 04/11/2017 10:00:00
Date and time modified: 05/03/2017 14:16:37
